# Gut Microbiota and Diabetes: Pioneering New Treatment Frontiers

**DOI:** 10.2174/0118715303342579241119155225

**Published:** 2025-01-08

**Authors:** Rupendra Shakya, Ponnurengam Malliappan Sivakumar, Pranav Kumar Prabhakar

**Affiliations:** 1 Department of Medical Laboratory Sciences, School of Allied Medical Sciences, Lovely Professional University, Panjab, 144001, India;; 2 School of Medicine and Pharmacy, Duy Tan University, Da Nang, Vietnam;; 3 Parul Institute of Applied Sciences and Research and Development Cell, Parul University, Vadodara, Gujarat, 391760, India

**Keywords:** Gut microbiota, dysbiosis, glycemic control, prebiotics, probiotics, symbiotic

## Abstract

Diabetes Mellitus (DM) is a complex metabolic disorder characterized by chronic hyperglycemia and poses significant global health challenges. Conventional treatments, such as insulin therapy and lifestyle modifications, have shown limited efficacy in addressing the multifactorial nature of DM. Emerging evidence suggests that gut microbiota, a diverse community of microorganisms critical for metabolism and immune function, plays a pivotal role in metabolic health. Dysbiosis, an imbalance in gut microbiota composition, has been linked to insulin resistance, obesity, and DM. Gut microbiota influences glucose metabolism through mechanisms, including short-chain fatty acid production, gut permeability regulation, and immune system interactions, indicating a bidirectional relationship between microbial health and metabolism. Clinical and experimental studies demonstrate that modulating gut microbiota through dietary interventions (prebiotics, probiotics, synbiotics) improves glycemic control and insulin sensitivity in DM patients. Fecal Microbiota Transplantation (FMT) has also shown promise in restoring healthy gut microbiota and alleviating DM-related metabolic disturbances. However, challenges remain, including the need for personalized treatments due to individual microbiota variability and the unknown long-term effects of these interventions. Future research should focus on elucidating the mechanisms by which gut microbiota influences metabolism and refining personalized approaches to enhance DM management.

## 1 INTRODUCTION

Diabetes Mellitus (DM) is a pervasive metabolic disorder that has emerged as one of the most formidable public health challenges of the 21^st^ century. Characterized by chronic hyperglycemia, DM results from defects in insulin secretion, insulin action, or both, leading to an array of complications that can severely impact the quality of life and longevity of an individual. This article provides an overview of the types, prevalence, global impact, current management strategies, and their limitations concerning DM. DM is primarily classified into three types: Type 1 Diabetes Mellitus (T1DM), Type 2 Diabetes Mellitus (T2DM), and Gestational Diabetes Mellitus (GDM) [1]. T1DM, also known as insulin-dependent diabetes, occurs due to the autoimmune destruction of the insulin-producing β-cells in the pancreas, leading to an absolute insulin deficiency [2]. It most commonly manifests in childhood or adolescence but can occur at any age. T2DM, the most prevalent form, arises from a combination of resistance to insulin action and an inadequate compensatory insulin secretory response. This type is strongly associated with obesity, sedentary lifestyles, and genetic factors and typically affects adults, although its incidence in children and adolescents is rising. GDM is a form of diabetes that is first recognized during pregnancy and is characterized by glucose intolerance with onset or first recognition during the second or third trimester.

The global prevalence of DM has been escalating at an alarming rate, attributed to aging populations, increasing obesity rates, urbanization, and lifestyle changes. According to the International Diabetes Federation, approximately 463 million adults were living with DM in 2019, and this number is projected to rise to 700 million by 2045 [3]. DM exerts a substantial burden on healthcare systems worldwide, with billions of dollars spent annually on treatment and management of the disease and its complications, which include cardiovascular diseases, kidney failure, neuropathy, and retinopathy, leading to blindness. The impact of DM extends beyond the individual, affecting economies through direct medical costs and indirect costs related to disability, loss of productivity, and premature mortality. In low- and middle-income countries, the rapid rise in DM prevalence is particularly concerning, straining resource-limited healthcare systems and hindering economic development.

### 1.1 Current Management Strategies

As diabetes mellitus is a lifestyle disease, it can be managed not only through proper medications but also through the change or modification of habits and living behaviors. The medicines that are available for the management of diabetes mellitus are broadly classified into two categories: insulin or oral hypoglycemic drugs. Most of these medicines are associated with some or more side effects. Management of DM focuses on controlling blood glucose levels to prevent or delay the onset of complications, requiring a multifaceted approach that includes lifestyle modifications, monitoring and managing blood glucose, and pharmacotherapy. For individuals with T1DM, lifelong insulin therapy is essential for survival. In contrast, management strategies for T2DM typically start with lifestyle interventions aimed at weight loss, increased physical activity, and dietary modifications, followed by the introduction of oral hypoglycemic agents or insulin when necessary [4]. Emerging management strategies also emphasize the importance of patient education, self-monitoring of blood glucose, and the use of technology, such as continuous glucose monitoring systems and insulin pumps, to enhance disease control. Despite advancements in treatment options, the management of DM remains a challenge due to the progressive nature of the disease and the need for individualized treatment plans.

### 1.2 Limitations of Current Management Strategies

Despite advancements in diabetes mellitus (DM) management, current strategies present notable limitations. A major challenge lies in achieving and sustaining adequate glycemic control, as many patients fail to reach or maintain target glucose levels due to the multifaceted nature of the disease, the adverse effects of pharmacological treatments, and the difficulties associated with long-term adherence to lifestyle modifications [5]. Furthermore, conventional therapies do not adequately target the underlying pathophysiological mechanisms of Type 2 Diabetes Mellitus (T2DM), particularly insulin resistance and β-cell dysfunction, leading to the progressive deterioration of β-cell function and subsequent disease progression. The risk of hypoglycemia, a potentially fatal complication, remains a critical concern with insulin therapy and certain oral hypoglycemic agents [6]. Additionally, the financial burden of DM management, especially the costs associated with insulin and glucose monitoring technologies, presents a significant barrier to effective treatment, particularly in low-resource settings. Moreover, individual variability in therapeutic response, influenced by genetic, environmental, and lifestyle factors, further complicates disease management [7]. The current one-size-fits-all approach is often insufficient, emphasizing the need for more personalized, targeted interventions that address the specific pathophysiological characteristics and risk profiles of each patient to optimize treatment outcomes.

Diabetes mellitus remains a significant global health issue, with its prevalence continuing to rise. While current management strategies have made strides in controlling the disease, significant limitations remain. These include challenges in achieving sustained glycemic control, the disease's progressive nature, the risk of hypoglycemia associated with certain treatments, and the economic burden of management. There is a critical need for continued research and innovation to develop more effective, personalized, and accessible treatment options for individuals living with DM [6]. Despite the growing body of research, there is a critical knowledge gap in finding treatment approaches that not only control symptoms but also address the root causes of DM. One emerging frontier in DM management is the modulation of gut microbiota, which has shown the potential to improve insulin sensitivity, reduce inflammation, and enhance metabolic health [8]. The gut microbiota represents a novel therapeutic target that could revolutionize how DM is treated, offering a more holistic, personalized approach. This review explores the role of gut microbiota in DM, evaluating its potential as a pioneering tool in the development of new treatment strategies that can overcome the limitations of current therapies. Addressing these challenges will require a concerted effort from healthcare providers, researchers, policymakers, and patients to mitigate the impact of this chronic disease on individuals and societies worldwide.

### 1.3 Gut Microbiota

The microbial ecosystem in the human gut is highly complex, with 1000-1500 distinct bacterial species and 10^13^-10^14^ distinct microorganisms that are essential to human health. Within this complex ecosystem, the most common species belong to the Clostridium group and Gram-positive bacteria [9]. The gastrointestinal system starts to colonize during delivery. The microbiota is more unstable and less diverse in the first two years of life than it is in adulthood, when complexity and diversity are higher. The age, gender, and genetic makeup of the host, in addition to several extrinsic variables like ambient conditions and hygiene, influence the composition of gut microbiota. However, the effect of nutrition is also evident. Certain food components, like vitamins, fatty acids, carbs, prebiotics, and probiotics, can change the composition of the gut microbiota [10]. Understanding the gut microbiota, an intricate ecosystem of microorganisms residing in the gastrointestinal tract, has revolutionized our approach to human health and disease. This complex community, comprising bacteria, archaea, viruses, and fungi, plays a pivotal role in maintaining physiological balance and has been linked to a myriad of health outcomes [10]. As experts delve deeper into the microbiota's secrets, it becomes clear that these microscopic inhabitants are fundamental to our well-being.

### 1.4 Composition of the Gut Microbiota

**Fig. (1):**
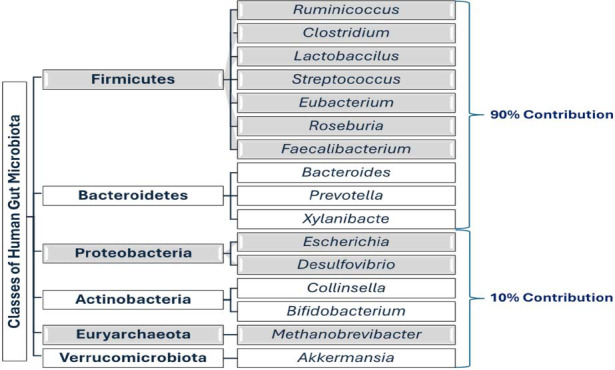
Six important classes of gut microbiome and some important species.

The human gut microbiota is a dynamic and diverse microbial community, with its composition influenced by various factors, including genetics, diet, environment, and age. The gut is predominantly colonized by bacteria, with Firmicutes and Bacteroidetes being the most prevalent phyla, followed by Actinobacteria, Proteobacteria, and Verrucomicrobia [11]. Each individual harbors a unique microbiota, a microbial fingerprint, yet certain patterns and functionalities are conserved across healthy populations. This microbial diversity is not random but rather a finely balanced ecosystem that performs essential functions for the host. The gut microbiota is established at birth and evolves throughout life, influenced by the mode of delivery, breastfeeding, antibiotic exposure, and diet [12]. A healthy, balanced gut microbiota is characterized by a high diversity of microbial species, which is associated with better health outcomes (Fig. **1**).

### 1.5 Functions of the Gut Microbiota

Understanding the functions of the gut microbiota offers insights into its critical contributions to the host's physiological, metabolic, and immune processes. The multifaceted roles of the gut microbiota encompass nutrient metabolism, maintenance of structural integrity of the gut mucosal barrier, immunomodulation, protection against pathogens, and modulation of the nervous system [13]. The gut microbiota performs several critical functions that are indispensable to human health. It plays a crucial role in the digestion and absorption of nutrients, particularly by breaking down complex carbohydrates that the host cannot digest, resulting in the production of short-chain fatty acids (SCFAs) like butyrate, propionate, and acetate [14]. These SCFAs serve as energy sources for colonocytes, regulate metabolism, and have anti-inflammatory effects. Moreover, the gut microbiota is integral to the development and function of the immune system. It stimulates the immune system to distinguish between pathogens and non-harmful antigens and modulates immune responses. A balanced microbiota is essential for maintaining the integrity of the gut barrier, preventing the translocation of harmful bacteria and substances into the bloodstream, a process crucial for avoiding systemic inflammation and infection.

- **Nutrient Metabolism and Energy Harvest:** One of the primary functions of the gut microbiota is to facilitate the digestion and absorption of nutrients, particularly those that the human digestive enzymes cannot process effectively. These microorganisms possess the enzymatic capacity to break down complex dietary polysaccharides and fibers into absorbable monosaccharides and SCFAs, such as acetate, propionate, and butyrate [15]. SCFAs serve as a significant energy source for colonocytes, contribute to lipogenesis and gluconeogenesis in the liver, and play a role in regulating lipid and glucose metabolism [16]. Furthermore, the gut microbiota is involved in the synthesis of essential vitamins, including vitamin K and certain B vitamins, which are critical for energy production, DNA synthesis, and maintenance of the nervous system [15].
- **Structural Integrity and Barrier Function:** The gut microbiota is integral to the development and maintenance of the gut's structural integrity. It stimulates the growth and proliferation of intestinal epithelial cells, influencing the turnover and repair of the gut lining. The microbiota also enhances the production of mucus by goblet cells, which serves as a physical barrier protecting the epithelial cells from pathogens and toxic substances [17]. Additionally, SCFAs, particularly butyrate, strengthen the barrier function by enhancing the assembly of tight junction proteins, thereby reducing gut permeability and preventing the translocation of harmful substances into the bloodstream [18].
- **Immunomodulation:** The gut microbiota plays a critical role in shaping the immune system, ensuring a balanced immune response that is robust against pathogens while maintaining tolerance to self-antigens and beneficial microorganisms. It influences the development and function of the gut-associated lymphoid tissue (GALT), which houses a significant portion of the body's immune cells [19]. The microbiota modulates the maturation and differentiation of various immune cells, including T cells, B cells, dendritic cells, and macrophages. It promotes the production of anti-inflammatory cytokines and regulatory T cells, which are crucial for maintaining immune tolerance and preventing autoimmune responses. Conversely, the microbiota can stimulate pro-inflammatory responses necessary for defending against pathogenic infections [20].
- **Protection against Pathogens:** The gut microbiota confers protection against invading pathogens through several mechanisms, collectively termed colonization resistance. The microbial community competes with pathogens for nutrients and attachment sites on the gut epithelium, hindering pathogen colonization. Certain commensal bacteria produce antimicrobial substances, such as bacteriocins, which inhibit the growth of harmful microorganisms. Additionally, the gut microbiota modulates the immune response of the host, enhancing the ability to fight infections [21]. Dysbiosis, or imbalance in the gut microbiota, can impair these protective mechanisms, increasing susceptibility to infections and disease.
- **Modulation of the Nervous System:** The gut microbiota communicates with the central nervous system through various pathways, including neural, endocrine, and immune-mediated mechanisms, collectively known as the gut-brain axis. This bidirectional communication network allows the gut microbiota to influence brain function and behavior, impacting stress responses, anxiety, depression, and pain perception [22, 23]. Microbial metabolites, such as SCFAs and tryptophan metabolites, can cross the blood-brain barrier and interact with the nervous system, affecting neuroinflammation, neurogenesis, and neurotransmission [24]. The gut microbiota also influences the production of gut hormones and neurotransmitters, such as serotonin, which plays a significant role in regulating mood and gastrointestinal motility.

The functions of the gut microbiota are diverse and critical for the host's health and well-being. By facilitating nutrient metabolism, maintaining gut integrity, modulating the immune system, protecting against pathogens, and influencing the nervous system, the gut microbiota plays a central role in maintaining physiological balance. Disruptions in the composition or function of the gut microbiota, known as dysbiosis, are associated with a wide range of diseases, including inflammatory bowel disease, obesity, diabetes, cardiovascular diseases, and neuropsychiatric disorders [10]. Understanding the complex interactions between the gut microbiota and the host opens new avenues for therapeutic interventions aimed at restoring microbial balance and promoting health. As research in this field advances, the potential for manipulating the gut microbiota to prevent and treat disease becomes increasingly apparent, highlighting the importance of these microscopic partners in our journey toward optimal health.

### 1.6 The Role of Gut Microbiota in Diabetes

**Fig. (2):**
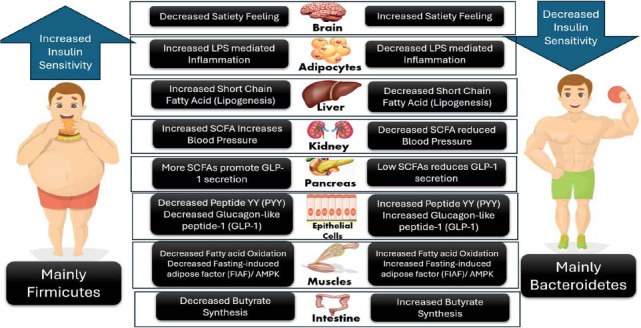
Possible mechanism through which the gut microbiota regulates insulin sensitivity.

The influence of gut microbiota on human health is profound and multifaceted. Beyond its essential roles in digestion and immunity, research has unveiled its involvement in regulating metabolism, protecting against pathogens, and even influencing mood and behavior through the gut-brain axis. Dysbiosis, an imbalance in the gut microbiota, has been associated with a range of health issues, including inflammatory bowel disease, obesity, T2DM, cardiovascular diseases, and even mental health disorders, such as depression and anxiety [10]. Intestinal incretin hormones, notably glucagon-like peptide-1 (GLP-1) and gastric inhibitory polypeptide (GIP), play a crucial role in managing postprandial blood glucose levels by enhancing insulin secretion from beta cells. The incretin effect, attributable to these hormones, accounts for 50-60% of insulin release following meal intake. GLP-1, beyond its insulinotropic function, also aids in reducing appetite, slowing gastrointestinal motility, and promoting beta cell proliferation, which is beneficial in treating obesity and T2DM [25]. However, the rapid degradation of incretin peptides by dipeptidyl-peptidase 4 limits their efficacy, leading to the exploration of therapeutic strategies that either mimic GLP-1 action or inhibit its breakdown (Fig. **2**).

Impaired incretin secretion is notably present in individuals with obesity and T2DM, signaling an early marker for these conditions. Therapeutic interventions targeting the incretin pathway, such as GLP-1R agonists and dipeptidyl-peptidase 4 inhibitors, have emerged, offering a new avenue for diabetes management [26]. Moreover, the role of gut microbiota in regulating incretin secretion has gained attention, with studies demonstrating that dietary interventions, particularly non-digestible carbohydrates like oligofructose, can enhance GLP-1 levels and improve metabolic outcomes. These effects are attributed to increased enteroendocrine L-cell numbers and are absent in GLP-1 receptor-deficient models, underscoring the contribution of gut microbiota to incretin-based therapies [27] (Fig. **2**).

Emerging research also highlights the direct regulation of incretin secretion by bacterial metabolites, such as hydrogen sulfide (H_2_S) and indole, produced by gut microbiota. These metabolites can either stimulate or inhibit GLP-1 release, indicating a complex interaction between gut microbes and incretin hormones [27]. Additionally, the gut microbiota influences incretin resistance and gastrointestinal motility, further complicating the understanding of incretin hormone dynamics.

SCFAs, produced by microbial fermentation of dietary fibers, are pivotal in energy metabolism and exhibit various beneficial effects, including enhanced GLP-1 secretion and improved insulin sensitivity (Fig. **2**). The modulation of SCFA levels through dietary interventions or probiotic administration represents a promising strategy for T2DM management [28]. Bacteroides are known for producing SCFAs, particularly propionate, which can enhance insulin sensitivity. These SCFAs stimulate the secretion of GLP-1 from the gut, which, in turn, promotes insulin secretion from the pancreas and improves glucose metabolism. Bacteroides also have anti-inflammatory properties, reducing systemic inflammation, which is closely associated with insulin resistance. In the kidneys, Bacteroides influence glucose reabsorption and sodium handling, indirectly affecting insulin sensitivity by modulating blood pressure and glucose levels. Firmicutes, especially when present in an imbalanced ratio with Bacteroides, have been linked to insulin resistance. They are more efficient at extracting energy from dietary sources, leading to increased production of SCFAs, particularly butyrate, which, while beneficial in moderate amounts, can contribute to energy overload and fat accumulation in excessive quantities. This energy excess is linked to the development of insulin resistance. Furthermore, an overabundance of Firmicutes can promote low-grade systemic inflammation by increasing gut permeability, allowing bacterial endotoxins like lipopolysaccharides (LPS) to enter circulation. This inflammation can impair insulin signaling pathways, particularly in the pancreas, by promoting β-cell dysfunction and reducing insulin secretion. In the kidneys, increased inflammation and oxidative stress may further contribute to insulin resistance through altered filtration and glucose handling. Furthermore, the interaction between bile acids and gut microbiota, involving bile acid metabolism genes, suggests a reciprocal relationship influencing metabolic health. Modifying gut microbiota composition to enhance bile acid metabolism may offer novel approaches for treating metabolic diseases.

Both Bacteroides and Firmicutes influence pancreatic β- cell function through their metabolic byproducts and the inflammatory milieu they create. Bacteroides-derived SCFAs promote GLP-1 secretion, enhancing β-cell function and insulin secretion, thereby improving insulin sensitivity. In contrast, an imbalance favoring Firmicutes increases systemic inflammation, which can damage β-cells, impairing insulin production and contributing to insulin resistance. The kidneys play a significant role in glucose homeostasis, particularly through glucose reabsorption in the proximal tubules. Bacteroides help regulate glucose levels by enhancing insulin sensitivity and reducing glucose reabsorption. In contrast, Firmicutes, especially in dysbiosis, may contribute to kidney inflammation and oxidative stress, exacerbating insulin resistance by impairing renal glucose handling. This can create a feedback loop of poor glucose control, further stressing the pancreas and worsening systemic insulin resistance.

Adipose tissue inflammation and function are also regulated by gut microbiota, which has implications for systemic metabolic health. Probiotic interventions have shown potential in reducing adipose tissue inflammation and promoting insulin sensitivity. Additionally, the “browning” of white adipose tissue, induced by gut microbiota modulation, holds promise for improving metabolic outcomes through increased energy expenditure and insulin sensitivity. Bile acids, acting as signaling molecules *via* FXR and TGR5 receptors, further link gut microbiota to metabolic regulation, offering insights into novel therapeutic targets for obesity and T2DM [29].

In conclusion, the intricate relationships between gut microbiota, incretin secretion, SCFA production, bile acid metabolism, and adipose tissue function underline the complex pathophysiology of metabolic diseases, such as T2DM. Understanding these interactions opens up new avenues for therapeutic interventions, emphasizing the potential of targeting the gut microbiota and its metabolic products to improve glycemic control and overall metabolic health.

### 1.7 Dysbiosis and Diabetes Mellitus

Emerging evidence suggests that the gut microbiota can influence drug metabolism and efficacy, highlighting its potential role in personalized medicine. The ability of microbiota to metabolize drugs can alter their pharmacokinetics and pharmacodynamics, affecting the therapeutic outcomes and side effects of medications.

Dysbiosis, defined as an imbalance in the microbial composition relative to a healthy state, plays a significant role in the pathogenesis of various diseases, including autoimmune and inflammatory conditions, central nervous system disorders, cancers, metabolic syndrome, T2DM, polycystic ovarian syndrome, and cardiovascular disease [10]. A crucial aspect of dysbiosis in the context of T2DM and metabolic syndrome is the altered ratio of Firmicutes to Bacteroidetes and a decreased abundance of *Akkermansia muciniphila*, which are linked to impaired glucose metabolism and obesity [30]. The predominance of Firmicutes over Bacteroidetes, often seen in obese individuals and those consuming high-calorie diets, correlates with negative metabolic outcomes. Conversely, increasing Bacteroidetes relative to Firmicutes can lead to weight loss and reduced inflammation.

*Akkermansia muciniphila,* a gram-negative bacterium residing in the gut mucosa, is notable for its beneficial effects on glucose and lipid metabolism and the promotion of intestinal immunity. This bacterium, by degrading mucin, plays a vital role in maintaining the gut barrier function, protecting against pathogens, and potentially altering the course of diabetes through its action on mucin secretion [30, 31]. Its presence or augmentation, through dietary interventions or direct administration, has been associated with improved metabolic markers in T2DM, highlighting its therapeutic potential [32]. Furthermore, dysbiosis contributes to increased inflammation and atherogenesis *via* gut microbial metabolites, such as trimethylamine-N-oxide (TMAO), derived from dietary choline. Elevated plasma TMAO levels are associated with atherosclerosis and have been implicated as a risk factor for cardiovascular disease, diabetes, and obesity. The role of gut microbiota in TMAO production underscores the complex interaction between diet, microbial metabolism, and disease risk [33]. The interaction between gut microbiota and host health extends to the modulation of gut hormones, notably through the production of SCFAs like butyrate, acetate, and propionate. These SCFAs influence insulin release and appetite regulation by increasing the secretion of GLP-1 and peptide YY (PYY), hormones that play crucial roles in glucose homeostasis and satiety [16, 33-35]. The mechanisms behind these effects involve the interaction of SCFAs with G-protein-coupled receptors on intestinal L cells, demonstrating a direct link between microbial metabolites and metabolic health.

The complex interplay between host molecules and the gut microbiota plays a pivotal role in the pathogenesis of T2DM, marked by dysbiosis or microbial imbalance. The regulation of gut microbial homeostasis involves several host proteins, including the protein deacetylase sirtuin 1, which shields high-fat diet (HFD)-fed mice from metabolic disorders by managing the Firmicutes and Bacteroidetes balance [36]. Conversely, the absence of sirtuin 1 leads to metabolic complications, such as adipose tissue hypertrophy and insulin resistance [37]. Zinc transporter 8 (ZnT8), enriched in pancreatic β-cells, influences diabetes development by altering intestinal morphology and the gut microbiota, thereby modulating fat accumulation and glucose tolerance [38]. Similarly, treatment with the farnesoid X receptor (FXR) agonist fexaramine in db/db mice enhances GLP-1 secretion and insulin sensitivity by modulating lithocholic acid-producing gut bacteria, highlighting the role of gut microbiota in glucose metabolism regulation [39]. Additionally, the knockout of tissue inhibitor of metalloproteinase 3 (Timp3) triggers liver steatosis and glucose intolerance due to induced dysbiosis and systemic inflammation *via* interleukin 6 (IL-6) signaling, with antibiotic treatment ameliorating these effects [40].

**Fig. (3):**
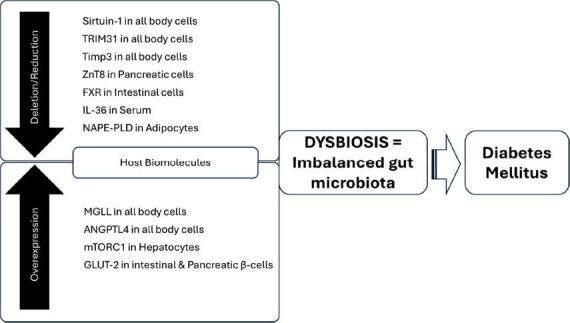
Dysbiosis caused by imbalanced host biomolecules results in diabetes mellitus.

Various host molecules, like IL-36, TRIM31, and NAPE-PLD, have been shown to influence metabolic health by reshaping the gut microbiota, thereby preventing obesity and T2DM. On the other hand, some proteins may promote T2DM by inducing gut microbiota dysbiosis [11]. For instance, resveratrol, an inhibitor of the mTORC1 pathway, ameliorates glucose intolerance and adjusts the microbial composition, suggesting the role of mTORC1 in glucose homeostasis and its connection to diabetic phenotypes through the gut microbiota. Similarly, monoglyceride lipase (MGLL) knockout mice display improved glucose tolerance and differential microbial responses to HFD, identifying MGLL as a potential metabolic disease target. Furthermore, the loss of angiopoietin-like 4 (ANGPTL4) and gut-specific deletion of glucose transporter 2 (GLUT2) also influence glucose metabolism through gut microbiota modulation [41]. These findings underscore the therapeutic potential of targeting host molecules like mTORC1, MGLL, ANGPTL4, and GLUT2 to manage T2DM, highlighting the critical role of the gut microbiota in metabolic health regulation and the necessity for further research to unravel these complex interactions [41] (Fig. **3**).

The therapeutic potential of modulating gut microbiota for T2DM treatment is supported by evidence that dietary fibers increase SCFA concentration, whereas high-fat diets reduce SCFA formation [42, 43]. Supplementation with SCFAs, particularly propionate, has been shown to reduce weight gain and increase the secretion of GLP-1 and PYY in overweight adults, offering a promising avenue for dietary intervention in metabolic disorders [44, 45]. Furthermore, the administration of SCFAs has been shown to raise plasma GLP-1 and PYY levels, further endorsing the role of gut microbiota in metabolic health. The dysregulation of the gut epithelial barrier, another consequence of dysbiosis, leads to increased absorption of LPS, a potent endotoxin from gram-negative bacteria. This disruption contributes to a chronic inflammatory state, elevating the risk of diabetes and insulin resistance. Thus, maintaining a healthy gut microbiota composition is crucial for preventing LPS-induced inflammation and metabolic dysfunction [44].

The ability of *Akkermansia muciniphila* to upregulate endogenous GLP-1 production, enhancing postprandial insulin secretion, signifies its importance in metabolic regulation [46, 47]. Decreased levels of this bacterium are associated with diabetes and obesity, while its increase through specific treatments correlates with improved metabolic outcomes. Recognized as a next-generation probiotic, *Akkermansia muciniphila* represents a focal point in the research for its potential to combat diabetes and obesity through gut microbiota modulation [46]. In summary, the intricate relationship of gut microbiota with metabolic diseases underscores the potential of microbiota-targeted therapies in managing T2DM and related conditions. By understanding and manipulating the gut microbial composition, particularly through the promotion of beneficial bacteria like *Akkermansia muciniphila* and the modulation of SCFA production, novel therapeutic strategies can be developed to improve metabolic health and reduce disease risk [48].

### 1.8 Role of Gut Microbiota in Kidneys of Patients with T2DM

The role of gut microbiota in kidney function, particularly in patients with T2DM, is increasingly recognized as a key factor in the progression of diabetic kidney disease (DKD). The gut microbiota influences kidney function through several mechanisms, including the modulation of systemic inflammation, production of metabolites, and regulation of metabolic pathways [49]. Following are the ways through which gut microbiota impacts kidney health in T2DM:

- **Modulation of Systemic Inflammation:** In T2DM, gut dysbiosis leads to increased intestinal permeability, often referred to as “leaky gut.” This allows bacterial endotoxins, such as LPS, to enter the bloodstream, triggering systemic inflammation. Chronic inflammation is a known contributor to insulin resistance and worsens kidney function by promoting oxidative stress, fibrosis, and endothelial dysfunction in renal tissues. Elevated levels of pro-inflammatory cytokines (*e.g.,* TNF-α, IL-6) have been associated with the progression of DKD.
- **Metabolite Production:** Gut bacteria, particularly Bacteroides and Firmicutes, produce SCFAs, such as acetate, propionate, and butyrate, which play a beneficial role in maintaining metabolic health. SCFAs can enhance insulin sensitivity and reduce inflammation, which indirectly supports kidney function by alleviating some of the metabolic stress linked to T2DM. However, in dysbiosis, the production of harmful metabolites, such as trimethylamine-N-oxide (TMAO) and p-cresyl sulfate, increases [50]. These uremic toxins accumulate in patients with kidney disease and contribute to renal inflammation, oxidative stress, and fibrosis, exacerbating DKD in T2DM patients.
- **Renal Glucose Handling and Sodium Reabsorption:** The gut microbiota influences renal glucose metabolism through SCFAs, which have been shown to regulate glucose homeostasis. SCFAs reduce glucose reabsorption in the kidneys by modulating sodium-glucose co-transporter 2 (SGLT2) activity, which plays a critical role in glucose handling in the proximal tubules [51]. In T2DM, where hyperglycemia is common, this process is disrupted, and the imbalance in SCFA production due to dysbiosis can worsen glucose reabsorption, further burdening the kidneys and promoting diabetic nephropathy.
- **Gut-Kidney Axis:** The concept of a gut-kidney axis refers to the bidirectional communication between the gut microbiota and kidney function. In T2DM, dysbiosis and the resulting metabolic endotoxemia contribute to the progression of kidney disease by promoting inflammation and fibrosis. Conversely, declining kidney function alters the composition of the gut microbiota, leading to increased production of nephrotoxic compounds, such as TMAO [52]. This creates a vicious cycle, where gut dysbiosis worsens kidney function and vice versa, accelerating the progression of DKD in T2DM patients.
- **Emerging Therapeutic Approaches:** Manipulating the gut microbiota through prebiotics, probiotics, and dietary interventions offers a promising therapeutic strategy for improving kidney function in T2DM patients. Restoring microbial balance by increasing beneficial bacteria that produce anti-inflammatory SCFAs may help mitigate renal inflammation and slow the progression of diabetic kidney disease [53]. There is also growing interest in the use of SGLT2 inhibitors, which not only improve glycemic control but also appear to have beneficial effects on both the gut microbiota and kidney function by reducing glucose reabsorption and modulating inflammatory pathways.

Gut microbiota plays a pivotal role in kidney function in patients with T2DM, primarily by influencing inflammation, metabolite production, and renal glucose handling. Therapeutic strategies aimed at modulating gut microbiota offer the potential to reduce the burden of diabetic kidney disease.

## CONCLUSION

The role of gut microbiota in T2DM development underscores its complex involvement in metabolic disease pathogenesis, extending our understanding beyond gut bacteria to include viruses and fungi. Studies reported that gut virus diversity is reduced in obese T2DM patients, and fungal communities differ significantly from healthy individuals, highlighting the intricate ecosystem of microbiota and its connection to T2DM. While research progresses, identifying specific microbes and underlying mechanisms remains a priority. Emerging antidiabetic strategies targeting the gut microbiota, such as probiotics and prebiotics, show clinical promise. For instance, *Lactobacillus rhamnosus* LRa05 has been found to lower fasting blood glucose and insulin resistance in T2DM mice by altering gut microbiota composition. Similarly, polysaccharides from Ganoderma lucidum improved insulin resistance and inflammation by correcting gut microbiota dysbiosis and enhancing intestinal barrier integrity. Additionally, traditional Chinese medicine and natural compounds, like ginsenoside Rg5, have been shown to modulate bacterial taxa beneficially, offering anti-diabetic effects. Non-pharmacological interventions, including bariatric surgery, fecal microbiota transplantation (FMT), diet, and exercise, also contribute to T2DM management by reshaping the gut microbiota. These findings suggest the pivotal role of gut microbiota in glucose metabolism, presenting innovative avenues for T2DM treatment and emphasizing the need for further research to explore this promising therapeutic target.

## LIST OF ABBREVIATIONS

DM: Diabetes Mellitus
FMT: Fecal Microbiota Transplantation
FXR: Farnesoid X Receptor
GALT: Gut-Associated Lymphoid Tissue
GDM: Gestational Diabetes Mellitus
GIP: Gastric Inhibitory Polypeptide
GLUT2: Glucose Transporter 2
IL-6: Interleukin 6
LPS: Lipopolysaccharides
SCFAs: Short-Chain Fatty Acids
SGLT2: Sodium-Glucose Co-Transporter 2
T1DM: Type 1 Diabetes Mellitus
